# Application of radio frequency based digital thermometer for real-time monitoring of dairy cattle rectal temperature

**DOI:** 10.14202/vetworld.2017.1052-1056

**Published:** 2017-09-12

**Authors:** Tridib Debnath, Santanu Bera, Suman Deb, Prasenjit Pal, Nibash Debbarma, Avijit Haldar

**Affiliations:** 1 ICAR Research Complex for North Eastern Hill Region, Tripura Centre, Agartala, Lembucherra - 799 210, Tripura, India; 2 Department of Livestock Production Management, West Bengal University of Animal and Fishery Sciences, Kolkata - 700 037, West Bengal, India; 3 Department of Computer Science and Engineering, National Institute of Technology, Agartala, Tripura, India; 4 Department of Extension and Social Sciences, College of Fisheries, Central Agricultural University, Lembucherra - 799 210, Tripura, India

**Keywords:** cattle, exercise, feeding, radio frequency device, rectal temperature, thermometer

## Abstract

**Aim:**

Dairy cattle health monitoring program becomes vital for detecting the febrile conditions to prevent the outbreak of the animal diseases as well as ensuring the fitness of the animals that are directly affecting the health of the consumers. The aim of this study was to validate real-time rectal temperature (RT) data of radio frequency based digital (RFD) thermometer with RT data of mercury bulb (MB) thermometer in dairy cattle.

**Materials and Methods:**

Two experiments were conducted. In experiment I, six female Jersey crossbred cattle with a mean (±standard error of the mean) body weight of 534.83±13.90 kg at the age of 12±0.52 years were used to record RT for 2 h on empty stomach and 2 h after feeding at 0, 30, 60, 90, and 120 min using a RFD thermometer as well as a MB thermometer. In experiment II, six female Jersey crossbred cattle were further used to record RT for 2 h before exercise and 2 h after exercise at 0, 30, 60, 90, and 120 min. Two-way repeated measures analysis of variance with *post hoc* comparisons by Bonferroni test was done.

**Results:**

Real-time RT data recorded by RFD thermometer as well as MB thermometer did not differ (p>0.05) before and after feeding/exercise. An increase (p<0.05) in RT after feeding/exercise in experimental crossbred cattle was recorded by both RFD thermometer and MB thermometer.

**Conclusion:**

The results obtained in the present study suggest that the body temperature recordings from RFD thermometer would be acceptable and thus RFD thermometer could work well for monitoring real-time RT in cattle.

## Introduction

The thermal physiology of animals is characterized by considerable, spatial and temporal variation in body temperature which is an excellent indicator of animal’s general health for assessing animal stress [1,2], warning for illness and diseases [3,4]. An animal health monitoring program to protect both animal and human is now the top priority job of animal owners after the incidence of bovine spongiform encephalopathy or mad cow disease in the USA on December 2003 [5]. The frequent epidemic outbreaks of foot-and-mouth disease (FMD) in domestic animals resulted in a huge economic loss in many countries [6-8]. An additional loss of $565 million was forecast for every hour delay of detection in a case study of a simulated outbreak of FMD in California, leaving a lesson that an early detection of any disease became a key function to alert farm workers for taking prompt and needful actions and thus preventing any massive economic damage from pandemic diseases in domestic animals [9].

The change in body temperature also provides information for identifying an animal with particular physiological conditions such as the onset of estrus [10] and approaching calving [11]. Hence, body temperature monitoring is a useful means for early detection of infectious diseases as well as physiological events in farm animals. Most commonly, rectal temperature (RT) is recorded and considered as body temperature by the dairy farmers in the detection and management of febrile conditions and changes in different physiological states of animals. However, capturing a sudden change in body temperature requires recording of body temperature in a continuous manner. The conventional method of recording RT in dairy animals with the help of mercury bulb (MB) thermometer is labor intensive and also costly. There is a need for solutions that provide continuous and automatic acquisition of this parameter. Surgically implanted integrated transponder tags or skin surface-mounted radio transmitters, data loggers [12-14] and infrared thermography [15,16] have been developed to record internal body temperature in animals. Considering animal welfare issue, external sensors such as neck collar, ankle ribbon, accelerometer, pedometer, and vibration sensor have been developed [17].

Of particular interest was the application of radio frequency identification device (RFID) that has already been used for determining automatically the physiological and behavioral activity as well as monitoring the health condition in human as well as livestock animals [18-20]. In view of monitoring animal health and ensuring animal well-being in the fast changing conditions of dairy farming, we hypothesized that radio - frequency based digital (RFD) thermometer could have similar RT recordings to that of the recordings of MB thermometer. The specific aim of this study was to determine if there was any change in RT recordings from an RFD thermometer as well as an MB thermometer after feeding and exercise in dairy cattle.

## Materials and Methods

### Ethical approval

The experimental protocol and animal care were met in accordance with the National guidelines for care and use of Agricultural Animals in Agricultural Research and Teaching as approved by the Ethical Committee for Animal Experiments of ICAR Research Complex for NEH Region, Barapani, Meghalaya, India.

### Experimental devices

RT was recorded by RFD thermometer as well as MB thermometer. In this study, DS18S20 Programmable Resolution1-Wire^®^ Digital Thermometer, Arduino Uno Model was applied for real-time monitoring of RT in experimental cattle. This RFD thermometer was developed and provided by Computer Science and Engineering Department, National Institute of Technology, Agartala, Tripura, India. The core functionality of the DS18B20 was its direct-to-digital temperature sensor. The resolution of the DS18B20 was configurable (9, 10, 11, or 12 bits), with the conversion of 12-bit temperature to digital word in 750 ms (maximum). Following the issuance of the Convert T [44h] command, a temperature conversion was performed, and the thermal data are stored in the scratchpad memory in a 16-bit, sign-extended two’s complement format. Information was sent to/from the device over a 1-wire interface so that only one wire was connected from a central microprocessor to the device. The power of reading, writing, and performing temperature conversions was derived from the data line itself without an external power source. It measured temperatures from −55°C to +125°C with an accuracy of 0.5°C from −10°C to +85°C.

### Experimental animals

**Figure-1:**
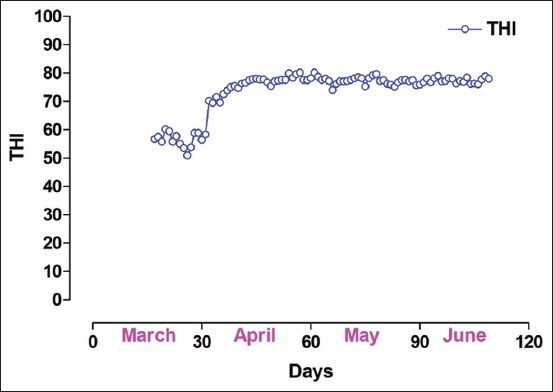
Temperature humidity index during the experimental period from 17^th^ March to 10^th^ June 2016.

Two experiments were conducted in female Jersey crossbred cattle to validate real-time RT data of RFD thermometer with RT data of MB thermometer at Livestock Farm of Indian Council of Agricultural Research (ICAR) Complex for North Eastern Hill (NEH) region, Lembucherra; Tripura, India, located 12.8 m above mean sea level at a 22°56^/^N latitude and 90°09^/^E longitude. The agro-climatic situation is humid, subtropical. Figure-1 shows temperature-humidity index (THI) during the experimental period from 17^th^ March to 10^th^ June 2016. THI remained below 60 during the first few days of the experiment and then varied between 70 and 80 indicating that the experimental animals were free from any severe environmental stress.

### Farm management

The experimental crossbred cattle were housed in well-ventilated individual pen with brick flooring and asbestos roofing and maintained in a sheltered paddock under natural day light and environmental conditions. The experimental sheds were cleaned and washed every day with an antiseptic agent to keep the animals free from any infection. They were fed according to the recommendations of the NRC [21] with an access to green grass/cut green leaves such as hybrid Napier, Congo signal and local grass, and commercially available concentrate feed. Clean drinking water was made available *ad libitum*. Deworming and vaccination against FMD and hemorrhagic septicemia were done as per standard schedule.

### Experiment I - Effect of feeding on RT in cattle

Based on the farm record, six non-pregnant Jersey crossbred cattle with a mean (±standard error of the mean [SEM]) body weight of 534.83±13.90 kg at the age of 12±0.52 years, was selected randomly from Livestock Farm of ICAR, Lembucherra, Tripura, India. The animals were checked clinically and confirmed that they were free from any anatomical, physiological or infectious disorders. The selected cattle were subjected to the feeding experiment individually. The experimental animals were fed 9:00 am and 4:00 pm and then kept on an empty stomach at night time for the experiment on the next day. On the day of the experiment, each animal was restrained in a crate under the shed at 9:00 am morning, and RT was recorded on an empty stomach at 30 min interval for 2 h starting from 9:00 am at 0, 30, 60, 90, and 120 min by inserting RFD thermometer as well as MB thermometer into the rectum. A gentle pressure was applied to ensure that the device touched the rectal wall. RT with MB thermometer was recorded for 1 min. In case of RFD thermometer, RT was recorded when there was no change in the reading for 3 s. Thereafter, feed and green fodder were offered to the experimental animal for 1 h, and again the RT was recorded at 0, 30, 60, 90, and 120 min by inserting RFD thermometer as well as MB thermometer into the rectum.

### Experiment II - Effect of exercise on RT in cattle

The previously selected six non-pregnant Jersey crossbred cattle were further used for the present experiment to capture the changes in RT both by RFD and MB thermometers due to the effect of exercise. Each animal was restrained in a crate under the shed at 9:00 am morning, and RT was recorded at 30 min interval for 2 h starting from 9:00 am at 0, 30, 60, 90, and 120 min using RFD thermometer as well as MB thermometer into the rectum. Thereafter, the experimental cattle were forced to exercise in a paddock for 1 h, and again RT was recorded in 0, 30, 60, 90, and 120 min using RFD thermometer as well as MB thermometer.

### Statistical analysis

All statistical analyses were performed using SAS 9.3 Statistical Software Package [22]. Data were presented in the text as the mean ± the SEM and presented graphically using graph pad PRISM 2.01 Software Package, 1995. To determine whether there was any significant difference between real-time RT data of RFD thermometer and RT data of MB thermometer before and after the treatment (feeding/exercise) as well as, find out the difference at each time point due to the effect of the treatment (feeding/exercise) in case of the RFD thermometer and the MB thermometer separately, both sets of temperature data collected at different time points were analyzed by two-way repeated measures analysis of variance (ANOVA). The *post hoc* comparison was done using the Bonferroni test.

## Results and Discussion

The present study was undertaken to validate real-time RT recordings from RFD thermometer as against the RT recordings of MB thermometer in cattle. The advent of RFID technology has the ability to produce real-time temperature readings which provide useful data to livestock producers for judging the physiological status of an individual animal. The result of repeated measure ANOVA for RT recorded by RFD thermometer and MB thermometer indicated that there was no significant difference (p>0.05) between real-time RT data recorded by RFD thermometer as well as MB thermometer either before (p=0.11) or after feeding (p=0.18) in this study. Similarly, no significant difference (p>0.05) between real-time RT data was recorded by RFD thermometer as well as MB thermometer before (p=0.56) and after exercise (p=0.51) in this study. To date, there is a dearth of information on the application of the RFD thermometer for real-time monitoring of dairy cattle RT. In earlier studies, peripheral body temperature readings from implantable radio frequency microchips were similar to RT readings in cattle [23]. Our recent study demonstrated the similar RT readings of RFD and MB thermometer in goats [24]. The present study also depicted that the real-time RT recordings of RFD thermometer did not vary to those RT recordings of MB thermometer in experimental cattle.

**Figure-2:**
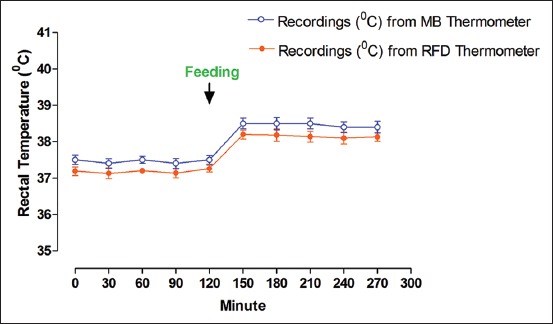
Changes of rectal temperature before and after feeding in crossbred cattle.

**Figure-3:**
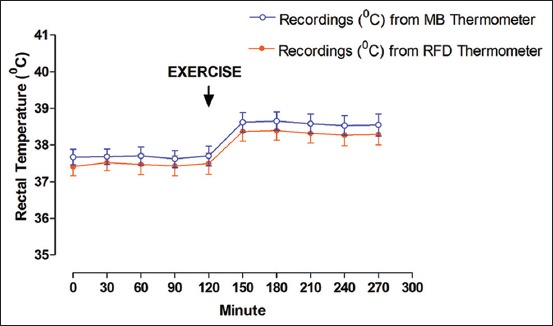
Changes of rectal temperature before and after exercise in crossbred cattle.

The changes of RT (mean±SEM) before and after feeding recorded by both RFD thermometer and MB thermometer are presented in Table-1 and Figure-2. A significant difference (p<0.05) in RT was recorded at various time points before and after feeding in experimental cattle. RT increased (p<0.05) at every time points due to the effect of feeding. Although it was non-significant (p>0.05) statistically, the recordings of RFD thermometer depicted an average of 0.28°C less temperature as compared to the readings of MB thermometer. The changes of RT (mean±SEM) before and after exercise recorded by both RFD thermometer and MB thermometer are presented in Table-2 and Figure-3. A significant difference (p<0.05) between RT was recorded at various time points before and after exercise in experimental cattle. RT increased (p<0.05) at every time points due to the effect of exercise. Although it was non-significant (p>0.05) statistically, the recordings of RFD thermometer depicted an average of 0.24°C less temperature as compared to the readings of MB thermometer. Of late, there is limited information on the effect of either feed intake or exercise on body temperature in animals. Previous studies have shown that body temperature increased after feed intake in rats [25] and human beings [26,27]. Few reports also depicted an increase in body temperature after exercise in human beings [28,29]. Recently, we have recorded an increase in RT recordings from both RFD and MB thermometers in goats [24]. In this study, we also recorded an increase in RT recordings from both RFD and MB thermometers after the feed intake/exercise in crossbred cattle. Furthermore, RFD thermometer and MB thermometer captured the similar pattern of the increasing trend of RT due to the effect of feeding/exercise in experimental cattle. Thus, this study indicated that RFD thermometer worked well for recording real-time RT in cattle.

**Table-1:** Changes of RT before and after feeding in crossbred cattle.

Time (min)	RT recorded by RFD thermometer		RT recorded by MB thermometer	
	
	Before feeding	After feeding	Before feeding	After feeding
0	37.19^b^±0.12	38.20^a^±0.12	37.50^b^±0.13	38.52^a^±0.15
30	37.13^b^±0.15	38.18^a^±0.17	37.40^b^±0.13	38.50^a^±0.17
60	37.20^b^±0.07	38.14^a^±0.15	37.47^b^±0.10	38.45^a^±0.15
90	37.14^b^±0.13	38.10^a^±0.16	37.43^b^±0.14	38.37^a^±0.15
120	37.26^b^±0.09	38.13^a^±0.12	37.50^b^±0.12	38.38^a^±0.16

^a,b^ Superscript indicate the significant difference of means at 5% level of significance. RT=Rectal temperature, MB=Mercury bulb

**Table-2:** Changes of RT before and after exercise in crossbred cattle

Time (min)	Body temperature recorded by RFD thermometer		Body temperature recorded by MB thermometer	
	
	Before exercise	After exercise	Before exercise	After exercise
0	37.41^b^±0.25	38.37^a^±0.26	37.67^b^±0.21	38.62^a^±0.27
30	37.52^b^±0.22	38.39^a^±0.26	37.68^b^±0.21	38.65^a^±0.25
60	37.47^b^±0.28	38.32^a^±0.27	37.70^b^±0.25	38.58^a^±0.27
90	37.43^b^±0.27	38.27^a^±0.29	37.62^b^±0.23	38.53^a^±0.27
120	37.49^b^±0.29	38.29^a^±0.29	37.70^b^±0.27	38.55^a^±0.30

^a,b^ Superscript indicate the significant difference of Means at 5% level of significance, RFD=Radio frequency based digital, MB=Mercury bulb

## Conclusions

RFD thermometer has been emerged as an effective instrument for recording and monitoring real-time RT in cattle. This RFD thermometer would allow dairy farmers for identifying febrile conditions, heat stress situation, productive and reproductive stages in dairy cattle in a timely manner. The present study directs to develop a wireless, non-invasive RFD thermometer connected with Android smartphone app using Bluetooth technology for enhancing the facility of real-time body temperature monitoring system round the clock.

## Authors’ Contributions

All authors contributed to conception and design of the study. TD carried out the research work as a part of M.V.Sc. thesis work. SB supervised the research work. SD developed the RFD thermometer. PP analyzed data. ND assisted in the research work. AH interpreted the results and drafted the article critically for important intellectual content. All authors read and approved the final manuscript.
